# Effects of tongue strengthening exercises on tongue muscle strength: a systematic review and meta-analysis of randomized controlled trials

**DOI:** 10.1038/s41598-022-14335-2

**Published:** 2022-06-21

**Authors:** Chien-Ju Lin, Yu-Shan Lee, Ching-Fang Hsu, Shu-Jung Liu, Jyun-Ying Li, Yin-Lan Ho, Hsin-Hao Chen

**Affiliations:** 1 Department of Family Medicine, Hsinchu MacKay Memorial Hospital, 690, Section 2, Guangfu Road, East District, Hsinchu City, 30071 Taiwan, ROC; 2 Department of Medical Library, MacKay Memorial Hospital, Tamsui Branch, New Taipei City, Taiwan, ROC; 3 Department of Physical Medicine and Rehabilitation, Hsinchu MacKay Memorial Hospital, Hsinchu City, Taiwan, ROC; 4 Department of Medicine, MacKay Medical College, New Taipei City, Taiwan, ROC; 5 MacKay Junior College of Medicine, Nursing, and Management, Taipei City, Taiwan, ROC

**Keywords:** Diseases, Health care, Medical research

## Abstract

Tongue strengthening exercise (TSE) has been proposed as an intervention to increase tongue strength and improve swallowing. However, clinical evidence of its effectiveness is lacking. In this review, seven databases were searched from inception to September 30, 2021 for randomized controlled trials that compared tongue strengths between the TSE intervention and control groups, obtained from maximal tongue elevation peak force in kilopascals (kPa). The Cochrane risk of bias tool was used for quality assessment. In total, 12 studies with 388 participants were included. The pooled meta-analysis demonstrated that the anterior tongue strength (ATS) (MD = 5.34 kPa; 95% CI 3.28–7.40; *I*^2^ = 71%) and posterior tongue strength (MD = 8.12; 95% CI 3.45–12.79; *I*^2^ = 90%) were significantly higher in the TSE intervention than that in the control group. Among healthy participants, subgroup analysis showed that TSE had improvements on ATS in all age groups, with the greatest improvement in old people (≥ 65 years) (MD = 8.01; 95% CI 4.39–11.64; *I*^2^ = 30%). Meta-regression analysis revealed a nonsignificant trend toward greater improvement on tongue strength with increasing TSE duration. This study provides positive evidence that TSE may be beneficial in improving tongue strength and could be applied for adults, especially healthy older adults.

## Introduction

Swallowing is composed of three phases: voluntary oral phase, involuntary pharyngeal phase, and involuntary esophageal phase. In the oral phase, the tongue plays an important role in food bolus mastication, formation, and transportation^1,2^. Weakness of the tongue muscle may result in dysphagia and increase the risk of aspiration. Therefore, adequate tongue muscle strength is essential for safe swallowing^1–3^.

Different from swallowing exercises that directly incorporate swallowing act, tongue strengthening exercise (TSE) is introduced as one of the isolated non-swallowing exercises^4^. Various types of TSE, i.e., isometric or isotonic training, unsupervised or supervised by trained therapist, and using a tongue depressor or using an electromyographic biofeedback devices, have been reported^5–12^. The standard protocol for TSE is not yet established to date.

The Iowa Oral Performance Instrument (IOPI) is currently the most commonly used device to assess the tongue muscle strength^5–8,10–12^. The air-filled silicone tongue bulb was positioned behind the central incisors, or behind the alveolar ridge to measure the maximal tongue elevation peak force (in kilopascals, kPa) as the anterior tongue strength (ATS) and then placed between the posterior tongue and hard-soft palate junction for the posterior tongue strength (PTS)^5–12^.

A previous meta-analysis demonstrated that TSE significantly improved the ATS and PTS in healthy adults and patients with dysphagia^13^. However, the authors only searched PubMed and Google Scholar and included English-only articles and studies without control groups in the meta-analysis^13^. Another systematic review investigating the effect of TSE on adult swallowing function indicated positive evidence for tongue muscle strength, but mixed results for swallowing safety and efficiency (using videofluoroscopic swallowing studies (VFSS), Penetration-Aspiration scale, transition duration, etc.)^14^. Recently, Lee et al.’s randomized controlled trial (RCT) showed that compared with the control group, the ATS in the TSE group was significantly increased; however, no significant difference was observed for the PTS in elderly adults^11^. Another RCT by Lazarus et al. revealed that TSE did not yield a statistically significant improvement in tongue strength in patients with oral and oropharyngeal cancer^7^. Therefore, this review and meta-analysis aimed to perform a comprehensive systematic review, including only RCTs to verify reported inconsistencies, and to evaluate the efficacy of TSE on the tongue strength to provide more convincing evidence.

## Methods

### Data sources and study selection

This systematic review adhered to the Preferred Reporting Items for Systematic Reviews and Meta-analyses (PRISMA) guidelines (Table S1)^15^. This review protocol was registered in the PROSPERO International Prospective Register of Systematic Reviews (CRD42021273739).

A librarian-mediated search of electronic databases (Cochrane, PubMed, Embase, International Clinical Trials Registry Platform, ClinicalTrials.gov, Cumulative Index to Nursing and Allied Health Literature, and PerioPath: Index to Taiwan Periodical Literature System) was conducted from inception to September 30, 2021, without language restrictions. Briefly, the following search terms were used: tongue, lingual, oral muscle, mouth exercise, resistance training, strengthening, and strength. References of relevant articles were also searched for potentially eligible studies. Full details of the search strategies are provided in Table S2.

After removing duplicates, two authors independently screened the titles and abstracts of each study and further reviewed the full texts to identify eligible studies. If a disagreement occurred, the corresponding author was consulted to achieve consensus. Studies were included if they met the following criteria: (1) participants aged ≥ 18 years, (2) studies that applied TSE, (3) studies including a control group without TSE, (4) studies reporting the tongue muscle strength at post-interventions, and (5) RCTs. Since there is still no universally accepted standard definition of TSE, tongue exercises with strength training including isometric/isotonic, unsupervised/supervised by a trained therapist, and using a tongue depressor/electromyographic biofeedback device were all included in our study. The exclusion criteria were as follows: (1) head-to-head comparisons of different tongue exercises without a control group; (2) training programs other than TSE or interventions combined with TSE, such as tongue hold swallowing and speech-language therapy; and (3) studies that did not report the tongue muscle strength as the outcome.

### Data extraction and quality assessment

Two authors independently extracted the following data from each included article: first author, year of publication, country of publication, number of enrolled participants, participant characteristics, applied TSE protocol, timing of outcome assessment, all outcome measurements, and main findings (Table S3). Any controversy was resolved by discussion with the third author. If a report was incomplete, the authors of the original study were contacted.

All studies that meet the inclusion criteria provided extractable data. Means and standard deviations for TSE and control groups were extracted. If the studies assessed the tongue strength at many time points, the baseline and the final post-intervention data were extracted. If intervention groups were divided into different training intensity groups, such as 100% of 1 repetition maximum (RM), 75% of 1 RM, and 50% of 1 RM, data were extracted from the 100% of 1 RM group.

Two reviewers independently assessed the risk of bias using the revised Cochrane risk of bias tool for randomized trials (RoB 2.0)^16^. This approach specifies three quality levels: (1) high, (2) some concerns, and (3) low. The following five domains were used in the assessment: (1) randomization process, (2) deviations from the intended interventions, (3) missing outcome data, (4) outcome measurement, and (5) selection of the reported results. Disagreements were resolved through discussions with the corresponding author.

### Statistical analysis

All statistical analyses and plotting were conducted using RStudio version 1.4.1106 (RStudio, Inc., Boston, MA, USA) with “meta” and “metafor” package^17^. The random-effects model was employed because the true effect could vary between studies. The pooled estimates of the mean difference (MD) and 95% confidence interval (CI) were calculated. To measure heterogeneity, Cochran’s Q-test and *I*^2^ statistic. A *p*-value of the Q test < 0.05 or *I*^2^ > 50% indicated the presence of heterogeneity were used^18^. Subgroup analysis was performed to evaluate the possible origins of heterogeneity. Meta-regression analysis was also conducted to investigate potential effect modifiers only when the data could be assessed throughout > 5 of the included studies^19^. Sensitivity analysis was performed by omitting each study to evaluate the stability of results. Finally, the risk of publication bias was assessed through funnel plot inspection and Egger’s test^20^.

## Results

### Search results and study characteristics

**Figure 1:**
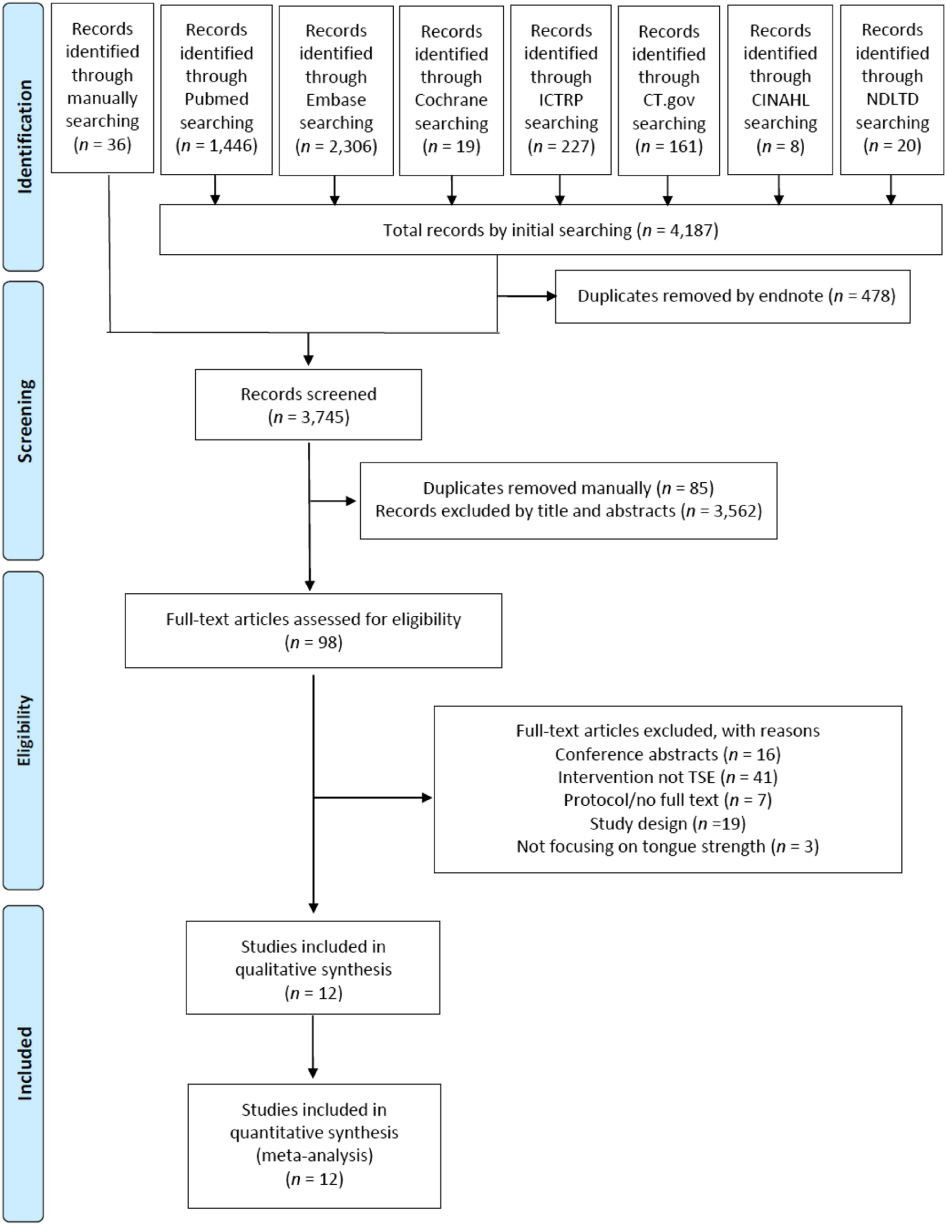
Flowchart of the study selection process. *ICTRP* International Clinical Trials Registry Platform, *CT.gov* ClinicalTrials.gov, *CINAHL* Cumulative Index to Nursing and Allied Health Literature, *NDLTD* the net worked digital library of theses and dissertations.

A total of 4187 articles were initially found in the initial database search, and additional 36 articles were found through the manual search. The initial database search uncovered 20 studies written in Chinese. Chinese is our native language, so we examined these studies without any translation method. After removing of duplicate articles and reviewing titles and abstracts, 98 full-text articles were retrieved and assessed for eligibility. Finally, 12 studies were included in our critical review and quantitative analysis^5–12,21–24^. The flow diagram of the study selection process is shown in Fig. 1.

All eligible studies were published from 2003 onward and were conducted in the United States (three studies), Korea (seven studies), Belgium and Taiwan. In total, 388 participants were included (199 and 189 in the TSE and control groups, respectively). Seven of 12 studies enrolled healthy participants^5,6,9–12,24^, whereas the other five studies focused on medical patients^7,8,21–23^. Among the studies involving healthy participants, three studies consisted of elderly people aged ≥ 65 years^9–11^. Three studies consisted of young adults^5,6,24^; and the last one study included both^12^. In the studies of medical patients, all participants had a diagnosis of cancer or stroke, with or without dysphagia^7,8,21–23^.

All included studies reported the ATS data, and six studies reported the PTS data^8,10–12,21,23^. The mean baseline ATS was 50.2 (range 36.8–66.3) kPa; and 29.7 (20.4–47.2) kPa for healthy participants and medical patients, respectively. The mean baseline PTS was 45.8 (32.2–53.5) kPa; and 23.9 (16.7–29.1) kPa for healthy participants and medical patients, respectively. Table S4 provides the list of baseline tongue strength of included studies.

The TSE intervention protocol varied in the total number of repetitions per day (calculated from the number of repetitions for each portion multiplied by the number of sets per day), frequency (3–5 days per week), and duration of intervention (lasting 4–8 weeks). Table S5 provided the summary of the training protocols of included studies. Most studies used the IOPI for collecting tongue strength, and one study by Park et al. used the TPS system (TPS 100, Cybermedic Inc, Iksan, South Korea)^9^. The TPS system, like IOPI, consisted of a tongue bulb with a pressure sensor, which could obtain the tongue pressure data. Table S3 depicts the detailed characteristics of included studies.

### Quality assessments

All included studies encountered “some concerns” using the RoB 2.0 tool for randomized trials. Although the participants or the therapist might be aware of the intervention, there was no deviations from intended intervention or deviation imbalance between groups. None of the included studies were deemed to be at high risk.

Eight studies had potential bias in the randomization process because of missing detailed allocation concealment^5,8–12,22,24^. Further, the prespecified analysis plan was unavailable for most studies, and that were rated “some concerns” in the domain of “selection of the reported result.” Table S6 exhibits the results of the full qualitative assessments.

### Effects of TSE on ATS

**Figure 2:**
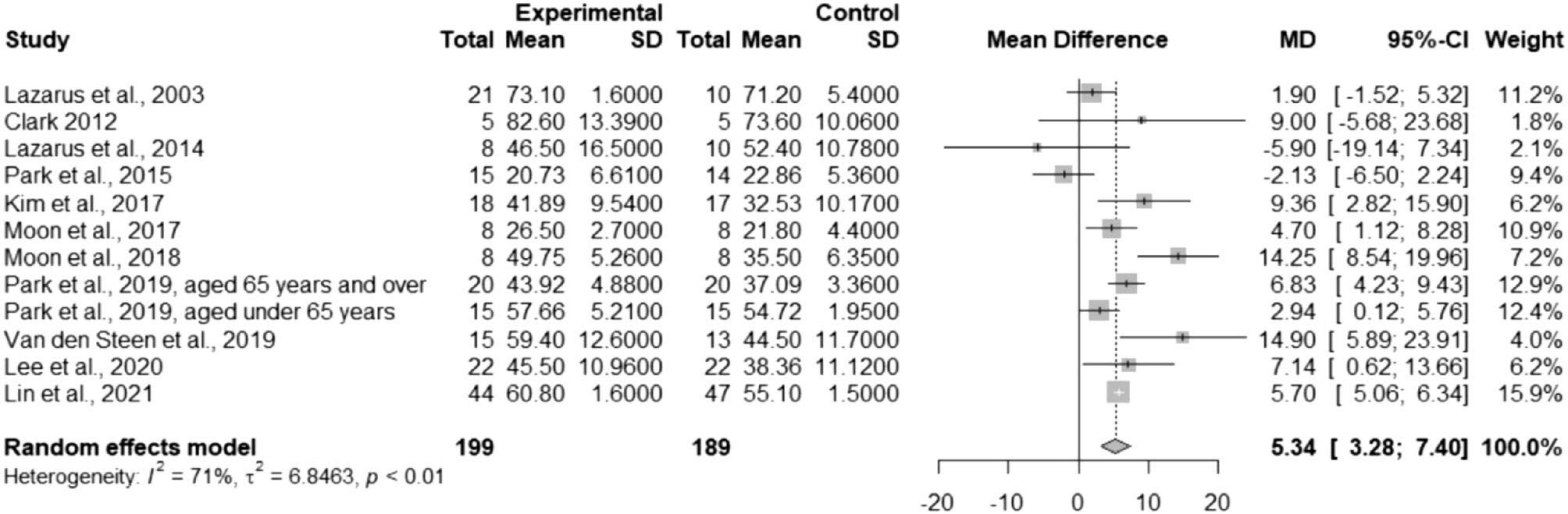
Forest plot of pooled anterior tongue strength after the intervention, comparing the tongue strengthening exercise group and the control group. *MD* mean difference, *CI* confidence interval.

To evaluate the effects of TSE on ATS, 12 studies were pooled in the meta-analysis. The results indicated that compared with the control group, the TSE group significantly increased ATS (MD = 5.34; 95% CI 3.28–7.40; *I*^2^ = 71%; between-study variance [τ^2^] = 6.85; Fig. 2).

**Figure 3:**
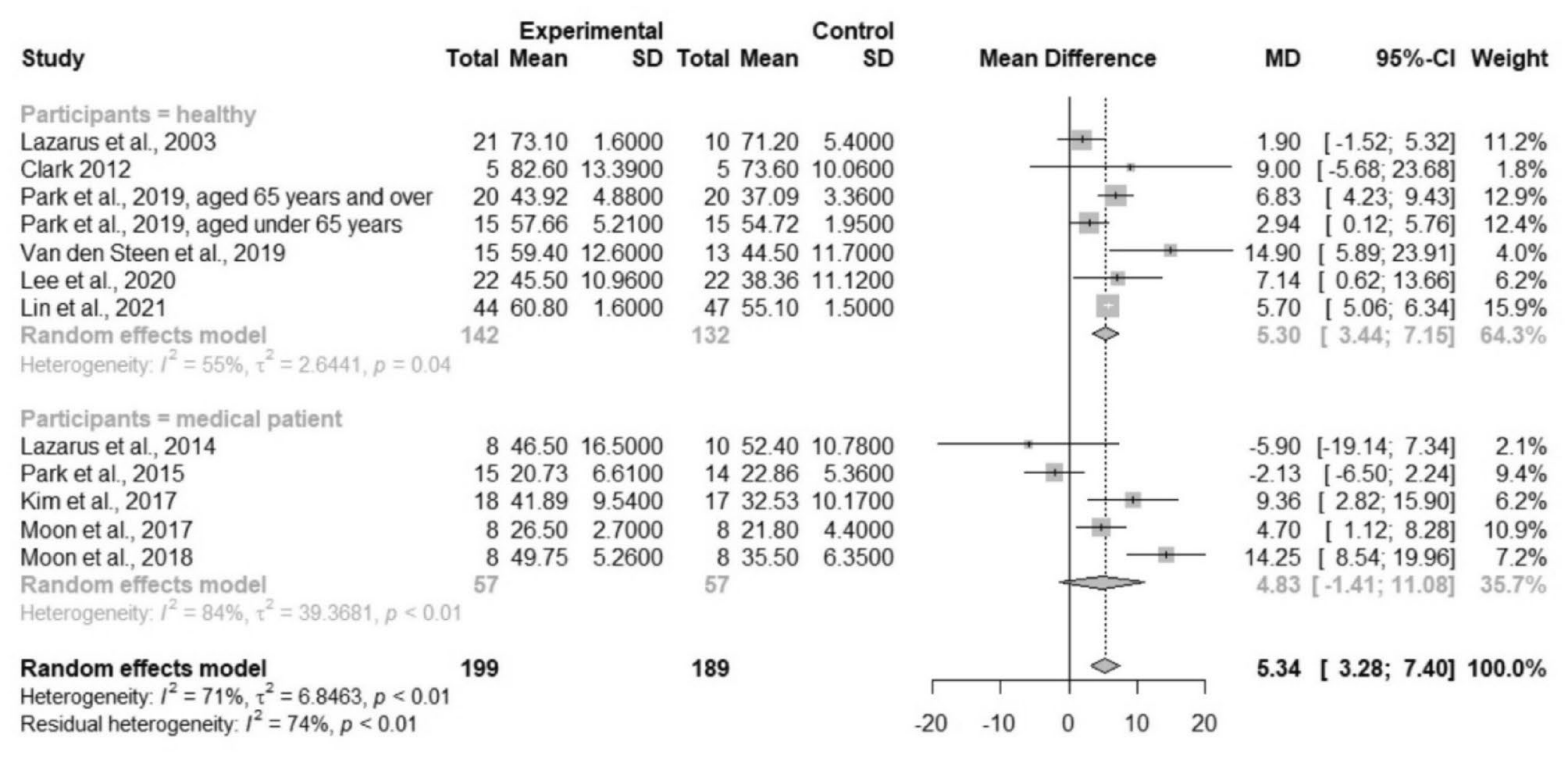
Forest plot of pooled anterior tongue strength after the intervention, comparing the tongue strengthening exercise group and the control group (subgroup analysis by the participants’ characteristics). *MD* mean difference, *CI* confidence interval.

**Figure 4:**
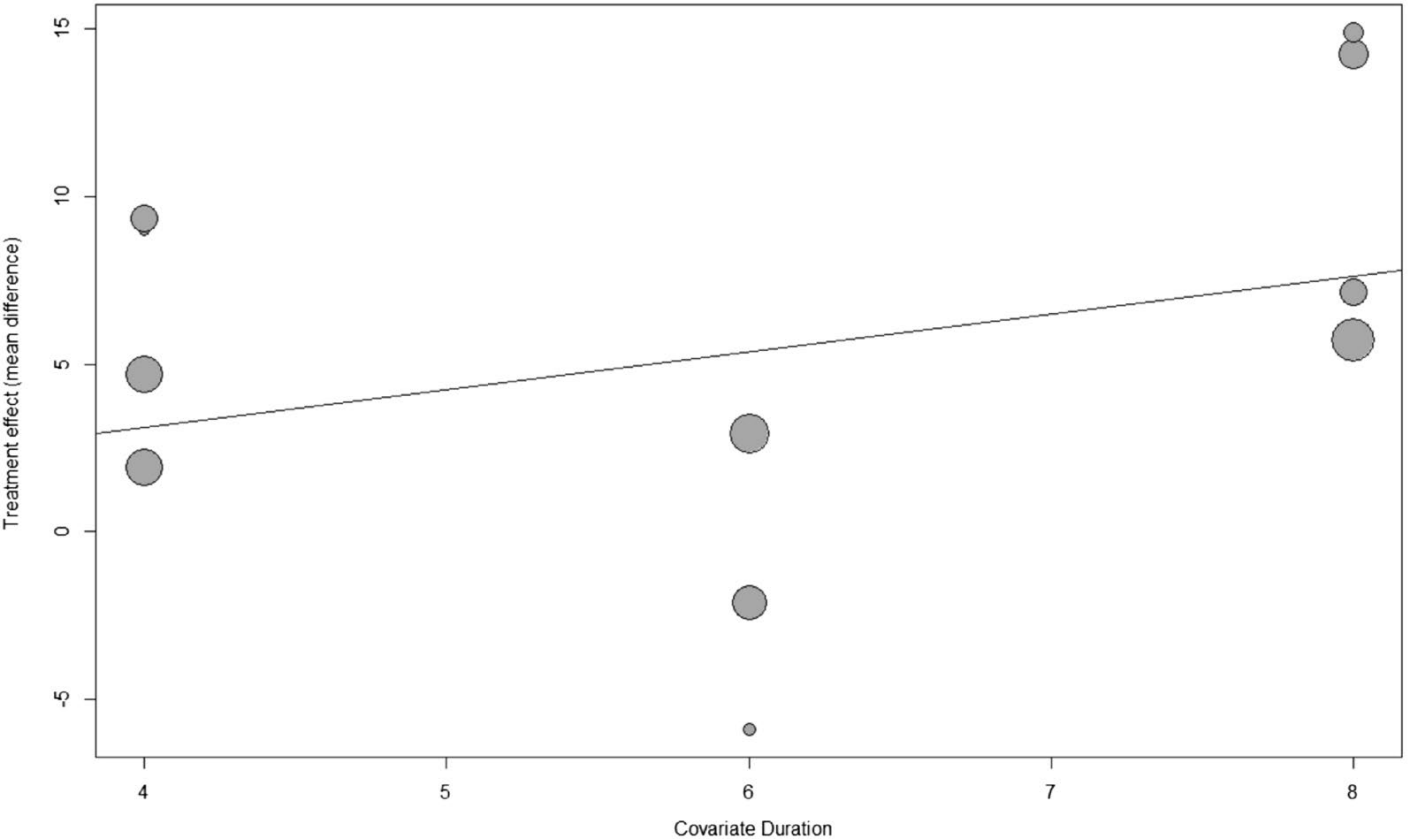
Meta-regression bubble plot of the correlation between effect of tongue strengthening exercise on anterior tongue strength and the duration of intervention. Each bubble represents a study and bubble size represents the sample size of the study.

Considering the heterogeneity in participants’ characteristics, subgroup analyses were performed. The results showed that the TSE group had significantly higher ATS in healthy participants (MD = 5.30; 95% CI 3.44–7.15; *I*^2^ = 55%; τ^2^ = 2.64), but not in medical patients (MD = 4.83; 95% CI − 1.41–11.08; *I*^2^ = 84%; τ^2^ = 39.37; Fig. 3). Another subgroup analysis based on age revealed that TSE had improvements on ATS in all age groups of healthy participants, with the greatest improvement in older adults (≥ 65 years) (MD = 8.01; 95% CI 4.39–11.64; *I*^2^ = 30%; τ^2^ = 3.66; Fig. S1). Univariate meta-regression analysis showed a nonsignificant trend for the increased treatment effect of TSE with increasing intervention duration (*p* = 0.27; Fig. 4). No evidence of effect modification by baseline ATS or the total number of repetitions per day was found (*p* = 0.76 and *p* = 0.79, respectively; bubble plots are shown in Figs. S2, S3, respectively).

Funnel plots and the Egger’s test indicated no significant publication bias (*p* = 0.89; Fig. S4). The sensitivity test showed robust results by omitting each study (Fig. S5).

### Effects of TSE on PTS

**Figure 5:**
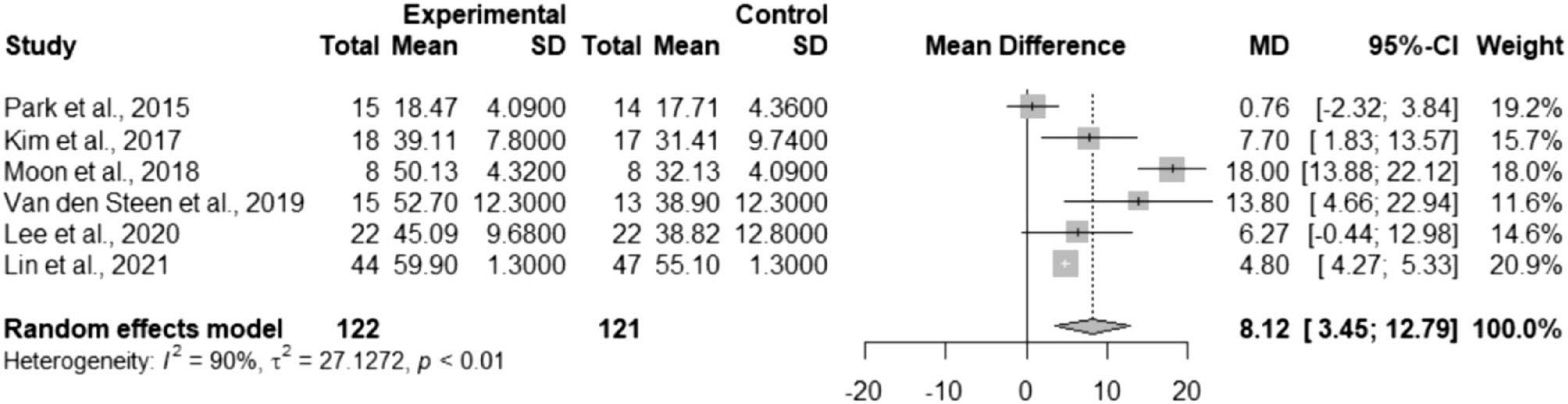
Forest plot of pooled posterior tongue strength after the intervention, comparing the tongue strengthening exercise group and the control group. *MD* mean difference, *CI* confidence interval.

**Figure 6:**
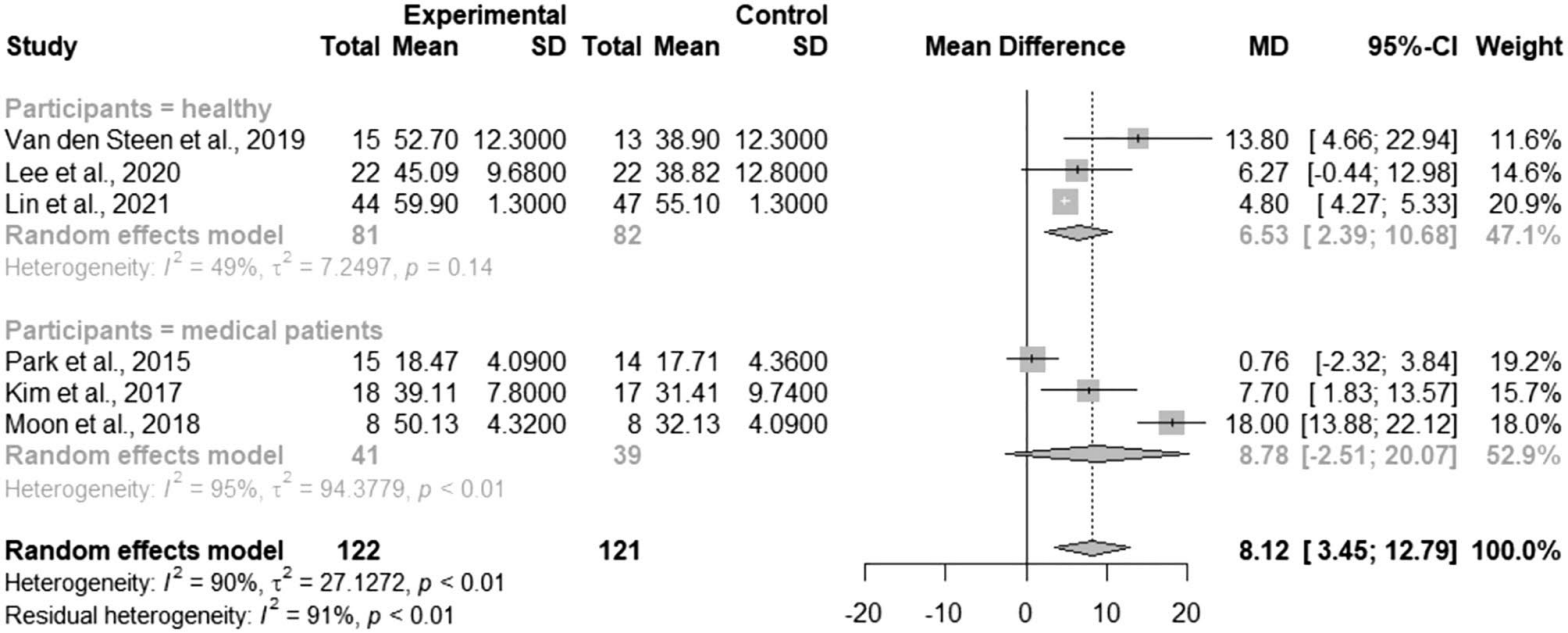
Forest plot of pooled posterior tongue strength after the intervention, comparing the tongue strengthening exercise group and the control group (subgroup analysis by the participants’ characteristics). *MD* mean difference, *CI* confidence interval.

Six studies reported PTS data and were included in the meta-analysis^8,10–12,21,23^. PTS in the TSE group was significantly more improved than that in the control group (MD = 8.12; 95% CI 3.45–12.79; *I*^2^ = 90%; τ^2^ = 27.13; Fig. 5). Subgroup analysis based on the participants’ characteristics revealed significant differences in healthy participants (MD = 6.53; 95% CI 2.39–10.68; *I*^2^ = 49%; τ^2^ = 7.25), but no statistically significant difference was observed in medical patients (MD = 8.78; 95% CI − 2.51–20.07; *I*^2^ = 95%; τ^2^ = 94.38; Fig. 6). The meta-regression showed a nonsignificant trend toward the increased treatment effects of TSE as the intervention duration increased (*p* = 0.49; Fig. S6). Baseline PTS or the total number of repetitions per day was not a significant effect modifier (*p* = 0.99 and *p* = 0.87, respectively; bubble plots are shown in Figs. S7, S8, respectively).

Although the funnel plots seemed asymmetrical by inspection, the Egger’s test indicated no significant publication bias (*p* = 0.38; Fig. S9). The sensitivity test confirmed the robustness of our results by omitting each study (Fig. S10).

## Discussion

This systematic review and meta-analysis showed that TSE significantly increased the ATS and PTS. Subgroup analysis revealed the significant benefits of TSE for healthy participants, but not for medical patients. Among healthy participants, our analysis found that TSE had improvements on ATS in all age groups, with the greatest degree of improvement in older adults aged ≥ 65 years. Furthermore, the meta-regression analysis revealed a nonsignificant trend toward an increased treatment effect of TSE as the intervention duration increased.

The present study indicated TSE is effective for both ATS and PTS, supporting the findings of previous literature reviews^14,25^. Previous studies revealed that the anterior tongue exhibits more type II muscle fibers, has a faster contraction response, and generates greater tongue strength while swallowing than the posterior tongue^26–29^. The posterior tongue contains a predominance of type I muscle fibers. They are slower, but more resistant to fatigue and provide sustained contractions, which plays a critical role for bolus propulsion into the pharynx^26–29^. Therefore, preserving and improving ATS and PTS are considered of paramount importance to reduce the risk of dysphagia.

A previous systematic review and meta-analysis reported that the tongue strength decreased with age, and ATS was typically stronger than PTS for healthy adults^30^, which was consistent with that of our study results. The study also demonstrated that tongue strength values seemed to be higher in healthy males than healthy females at the same age^30^. Another meta-analysis found that the influence of sex on tongue strength was only observed in individuals younger than 60 years but not in older individuals^31^. All studies we included analyzed the values of males and females together. Further studies considering the interaction of sex and age in tongue strength are warranted.

In medical patients, different disorders had various reductions on tongue strength^30^. In our included studies, one study enrolled patients with oropharyngeal cancer^7^, and four studies focused on patients with stroke^8,21–23^. Both the mean baseline ATS and PTS of medical patients were lower than healthy participants.

Subgroup analysis based on participants’ characteristics found that TSE significantly increased ATS and PTS in healthy participants, and the heterogeneity was reduced. Further subgroup analysis based on age revealed a greater improvement on ATS in healthy older adults. We assumed that different baseline tongue strength may have different responses to the intervention. As mentioned, tongue strength declined with age. The mean baseline ATS of three studies involving healthy young people was 66.1^5^, 66.3^6^, and 53.0 kPa^24^, respectively. However, the mean baseline ATS for studies involving healthy older adults ranged from 36.8 to 39.9 kPa^9–11^. Our meta-regression analysis also showed a nonsignificant trend for less treatment effect with higher baseline ATS. Therefore, we proposed that as tongue strength decreased with age, TSE may be more beneficial.

High heterogeneity and nonsignificant improvement were noted among medical patients because of some possible explanations. First, the number of RCTs focusing on this specific population was limited. Second, the control group in studies involving medical patients received traditional dysphagia therapy, such as effortful swallowing and Mendelsohn maneuver, which may influence the tongue strength^32,33^. Third, the disease severity and intervention timing may lead to greater heterogeneity. Lazarus et al.,’s study enrolled patients with oral and oropharyngeal cancer of different stages and different primary tumor locations^7^. In addition, some of included patients had dysphagia, but some did not. The other four studies enrolled dysphagia patients with different types of strokes (hemorrhagic/ischemic and right hemisphere/left hemisphere)^8,21–23^. A previous review demonstrated that the motor cortices control swallowing bilaterally but asymmetrically. Lesions at the left periventricular white matter may be more disruptive to swallowing behavior than those on the right^34^. Furthermore, the mean TSE intervention time since the stroke onset was 6.5^8^, 6.3^22^, 5.1^21^, and 2.1 months^23^, respectively. Previous studies indicated that dysphagia occurred in an average of 50% of stroke survivors, most of these patients recovered spontaneously, and 10 to 30% may have prolonged dysphagia up to 3 to 6 months^34–36^. The optimal timing to initiate rehabilitation after a stroke remains unknown. A review article demonstrated that early intervention has shown some promise for dysphagia, but only three studies were included, and the high proportion of spontaneous recovery made it difficult to assess the true impact of early rehabilitation^36^. Ultimately, intervention strategy might be of paramount importance for this specific population. Patients in the Lazarus et al.,’s study performed self-exercise using the tongue depressor, while the other four studies used the biofeedback device under the supervision of the therapist. It is difficult to say that the participants performed the exercise properly since the exercise performances (compliance) of the experimental and control groups were very different. This might explain that both groups showed no effect regardless of the participants in the Lazarus et al.,’s study. Further studies are warranted to investigate the effects of TSE on the tongue strength in medical patients.

Previous studies found the tongue strength during swallowing is lower than the maximal tongue strength and depends on bolus viscosity^25,29,37^. In brief, foods of higher viscosity required greater tongue pressure during swallowing than foods of lower viscosity. Previous studies revealed that healthy participants required approximately half of their maximum tongue pressure during liquid swallows^25,29,37^. Therefore, ATS and PTS, which were measured from the maximum tongue strength in all included studies could not reflect the true value of tongue pressure during swallowing. Despite previous studies and our hypothesis that tongue swallowing pressure was correlated with maximum tongue strength, it remains to be determined whether there is any evidence that TSE could generalize to the submaximal dynamic task of swallowing. For this reason, some studies using other outcome measures aimed to investigate the effects of TSE on the swallowing function. Two studies used videofluoroscopic dysphagia scale (VDS), a functional assessment scale consisting of 14 items, based on a videofluoroscopic swallowing study. The total score ranges from 0 to 100 points, with higher scores indicating severer dysphagia. One study showed that the TSE group had significantly improved VDS compared to the control group^21^; however, no statistically significant difference was observed between the two groups in another study^8^. Except for the VDS, various types of scales were used in different studies, such as the oropharyngeal swallow efficiency score, Mann assessment of swallowing ability, swallowing quality-of-life questionnaire, and oral health impact profile-14^7,8,11,21,23^. Due to the limited data, quantitative analysis was not conducted.

A previous systematic review investigating the effect of TSE on swallowing function reviewed seven articles (only two studies were RCT design)^14^. Five of the seven studies reported Penetration–Aspiration scale as a swallowing safety outcome, three studies showed significant improvement after TSE intervention^5,21,38^, but two studies found no statistically significant change^39,40^. Three studies documented the duration of swallowing, and only one study found that TSE improved oral transit duration and pharyngeal response duration for the 3-mL liquid bolus condition^39^. Limited studies and lack of standardized outcome measures lead to a particular barrier to data synthesis. High-quality RCTs with larger sample sizes are needed to clarify the evidence for the efficacy of TSE on swallowing function.

A previous review indicated that as training duration increases, tongue strength gradually increases. They found that the plateau was not reached from baseline to 8 weeks^13^. Our meta-regression showed that the effect of TSE on ATS and PTS did not significantly increase by increasing the duration of intervention. However, none of the included studies used the training program for > 8 weeks. Thus, the duration of plateau and sufficient training remains controversial. Our result demonstrated that the total number of repetitions per day was not a significant effect modifier. The possible explanation is differences in the contraction time. In three studies, the contraction time for each action was 2 s^5,7,8^, but was at least 10 s in Lin et al.’s study^12^. Moreover, some studies did not mention these details. Therefore, we expect that future studies providing more information about exercise variables such as frequency, intensity, the number of sets per day, inter-set rest interval, the number of repetitions per set, and the contraction time of each action will facilitate comparisons between studies.

With regards to the instruments of TSE, one study did not use a device for TSE^9^, and two studies conducted TSE using the tongue depressor^5,7^, which is easily accessible and inexpensive. Other studies used the biofeedback device during training, which is also widely used in the current practice. In our review, none of the included studies documented the side effects of TSE. Although TSE may be considered a safe intervention, it still needs to be used with caution in clinical application. In addition to the efficacy of TSE, safety issues are also an essential part of future studies.

As mentioned earlier, none of the included studies were rated at high risk of bias assessment. All included studies were rated “some concerns” because of missing detailed allocation concealment or prespecified analysis protocol. However, there were some potential risks of bias of the included studies might not be covered by the five domains of Rob 2.0 tool. First, only two of the 12 studies reported the sample size calculations^11,12^. Other studies mentioned small sample size as a limitation without further explanation; thus, the optimal sample size to detect statistical significance remains unclear. Second, most of the included studies were funded^5–7,9–12,23,24^, which might have led to more favorable efficacy outcomes. In Cochrane, the debate over whether funding is a source of bias is inconclusive^41^.

To the best of our knowledge, this is the first systematic review and meta-analysis of RCTs illustrating the treatment effects of TSE on tongue strength. We only included RCTs for meta-analysis to provide more reliable evidence. The potential impact of relevant effect modifiers such as the baseline tongue strength, and different exercise protocols was also explored. However, this study has several limitations. First, some trials had relatively small sample sizes; moreover, studies targeting specific population, such as young adults and medical patients were limited. Second, although a recent review article used a cut-off of < 5 studies to identify meta-regression analyses at risk of overfitting^19^, the Cochrane Handbook suggests a minimum of 10 studies per examined covariate in the meta-regression^42^. More studies are warranted to minimize the risk of overfitting and explore other potential effect modifiers. Third, most included studies were performed in the anterior and posterior tongue isometric strengthening training, and data of 100% of 1 RM were extracted for analysis. There are various types of tongue exercises, thus, future investigations focusing on different intensity or different parts of the tongue are warranted. Ultimately, most included studies only reported ATS or PTS results, indicating the strength of tongue elevation. However, the pressures in the lateral side and tongue strength during swallowing are also considered as important parameters. In addition to the tongue strength, further studies involving more comprehensive dysphagia assessment of dysphagia may help us better understand the clinical application of TSE.

## Conclusions

This updated review indicates that tongue strengthening training may have a beneficial effect on tongue strength. Positive evidence was found in healthy participants, with the greatest improvement in healthy older adults. Future research should focus on more assessment of swallowing function and investigation of the clinical rationale for use of TSE in medical patients.

## Supplementary Information

Supplementary Information.

## Data Availability

The datasets used and/or analyzed in this study are available from the corresponding author on reasonable request.
