# Supplementary material for: Effects of tongue strengthening exercises on tongue muscle strength: a systematic review and meta-analysis of randomized controlled trials
Source: Sci Rep. 2022 Jun 21;12:10438. doi: 10.1038/s41598-022-14335-2 (PMC9213467; doi:10.1038/s41598-022-14335-2)

Supplementary Materials

Effects of Tongue Strengthening Exercises on Tongue Muscle Strength: A Systematic Review and Meta-Analysis of Randomized Controlled Trials

Chien-Ju Lin[1], Yu-Shan Lee[1], Ching-Fang Hsu[1], Shu-Jung Liu[2], Jyun-Ying Li[3], Yin-Lan Ho[3], Hsin-Hao Chen[1,4,5*]

[1]Department of Family Medicine, Hsinchu MacKay Memorial Hospital, Hsinchu City, Taiwan

[2]Department of Medical Library, MacKay Memorial Hospital, Tamsui Branch, New Taipei City, Taiwan

[3]Department of Physical Medicine and Rehabilitation, Hsinchu MacKay Memorial Hospital, Hsinchu City, Taiwan

[4]Department of Medicine, MacKay Medical College, New Taipei City, Taiwan

[5]MacKay Junior College of Medicine, Nursing, and Management, Taipei City, Taiwan

[*]Corresponding author: Dr. Hsin-Hao Chen, Department of Family Medicine, Hsinchu MacKay Memorial Hospital, No. 690, Section 2, Guangfu Road, East District, Hsinchu City 30071, Taiwan. Tel.: +886-3-688-9595; Email addresses: 2033@mmh.org.tw

Table S1. PRISMA checklist

Section and Topic	Item #	Checklist item	Location where item is reported
**TITLE**			
Title	1	Identify the report as a systematic review.	p.1
**ABSTRACT**			
Abstract	2	See the PRISMA 2020 for Abstracts checklist.	p.2
**INTRODUCTION**			
Rationale	3	Describe the rationale for the review in the context of existing knowledge.	p.3-4
Objectives	4	Provide an explicit statement of the objective(s) or question(s) the review addresses.	p.3-4
**METHODS**			
Eligibility criteria	5	Specify the inclusion and exclusion criteria for the review and how studies were grouped for the syntheses.	p.4-5
Information sources	6	Specify all databases, registers, websites, organisations, reference lists and other sources searched or consulted to identify studies. Specify the date when each source was last searched or consulted.	p.4
Search strategy	7	Present the full search strategies for all databases, registers and websites, including any filters and limits used.	Table S2
Selection process	8	Specify the methods used to decide whether a study met the inclusion criteria of the review, including how many reviewers screened each record and each report retrieved, whether they worked independently, and if applicable, details of automation tools used in the process.	p.5-6
Data collection process	9	Specify the methods used to collect data from reports, including how many reviewers collected data from each report, whether they worked independently, any processes for obtaining or confirming data from study investigators, and if applicable, details of automation tools used in the process.	p.5-6
Data items	10a	List and define all outcomes for which data were sought. Specify whether all results that were compatible with each outcome domain in each study were sought (e.g. for all measures, time points, analyses), and if not, the methods used to decide which results to collect.	p.5-6, table S3
	10b	List and define all other variables for which data were sought (e.g. participant and intervention characteristics, funding sources). Describe any assumptions made about any missing or unclear information.	p.5-6, table S3

Section and Topic	Item #	Checklist item	Location where item is reported
Study risk of bias assessment	11	Specify the methods used to assess risk of bias in the included studies, including details of the tool(s) used, how many reviewers assessed each study and whether they worked independently, and if applicable, details of automation tools used in the process.	p.6
Effect measures	12	Specify for each outcome the effect measure(s) (e.g. risk ratio, mean difference) used in the synthesis or presentation of results.	p.6
Synthesis methods	13a	Describe the processes used to decide which studies were eligible for each synthesis (e.g. tabulating the study intervention characteristics and comparing against the planned groups for each synthesis (item #5)).	p.6
	13b	Describe any methods required to prepare the data for presentation or synthesis, such as handling of missing summary statistics, or data conversions.	p.6
	13c	Describe any methods used to tabulate or visually display results of individual studies and syntheses.	p.6
	13d	Describe any methods used to synthesize results and provide a rationale for the choice(s). If meta-analysis was performed, describe the model(s), method(s) to identify the presence and extent of statistical heterogeneity, and software package(s) used.	p.6
	13e	Describe any methods used to explore possible causes of heterogeneity among study results (e.g. subgroup analysis, meta-regression).	p.6
	13f	Describe any sensitivity analyses conducted to assess robustness of the synthesized results.	p.6
Reporting bias assessment	14	Describe any methods used to assess risk of bias due to missing results in a synthesis (arising from reporting biases).	p.5-6
Certainty assessment	15	Describe any methods used to assess certainty (or confidence) in the body of evidence for an outcome.	p.6
**RESULTS**			
Study selection	16a	Describe the results of the search and selection process, from the number of records identified in the search to the number of studies included in the review, ideally using a flow diagram.	p.7, figure 1
	16b	Cite studies that might appear to meet the inclusion criteria, but which were excluded, and explain why they were excluded.	figure 1
Study characteristics	17	Cite each included study and present its characteristics.	Table S3

Section and Topic	Item #	Checklist item	Location where item is reported
Risk of bias in studies	18	Present assessments of risk of bias for each included study.	Table S5
Results of individual studies	19	For all outcomes, present, for each study: (a) summary statistics for each group (where appropriate) and (b) an effect estimate and its precision (e.g. confidence/credible interval), ideally using structured tables or plots.	Table S3
Results of syntheses	20a	For each synthesis, briefly summarise the characteristics and risk of bias among contributing studies.	p.7-10
	20b	Present results of all statistical syntheses conducted. If meta-analysis was done, present for each the summary estimate and its precision (e.g. confidence/credible interval) and measures of statistical heterogeneity. If comparing groups, describe the direction of the effect.	p.7-10, figure 2, 4
	20c	Present results of all investigations of possible causes of heterogeneity among study results.	p.9-10, figure 3, S1-S4, S7-S10
	20d	Present results of all sensitivity analyses conducted to assess the robustness of the synthesized results.	p.9-10, figure S6, S12
Reporting biases	21	Present assessments of risk of bias due to missing results (arising from reporting biases) for each synthesis assessed.	p.9-10, figure S5, S11
Certainty of evidence	22	Present assessments of certainty (or confidence) in the body of evidence for each outcome assessed.	p.9-10
**DISCUSSION**			
Discussion	23a	Provide a general interpretation of the results in the context of other evidence.	p.10-15
	23b	Discuss any limitations of the evidence included in the review.	p.14-15
	23c	Discuss any limitations of the review processes used.	p.14-15

Section and Topic	Item #	Checklist item	Location where item is reported
	23d	Discuss implications of the results for practice, policy, and future research.	p.10-15
**OTHER INFORMATION**			
Registration and protocol	24a	Provide registration information for the review, including register name and registration number, or state that the review was not registered.	p.4
	24b	Indicate where the review protocol can be accessed, or state that a protocol was not prepared.	p.4
	24c	Describe and explain any amendments to information provided at registration or in the protocol.	p.4
Support	25	Describe sources of financial or non-financial support for the review, and the role of the funders or sponsors in the review.	p.21
Competing interests	26	Declare any competing interests of review authors.	p.21
Availability of data, code and other materials	27	Report which of the following are publicly available and where they can be found: template data collection forms; data extracted from included studies; data used for all analyses; analytic code; any other materials used in the review.	p.23

Table S2. Search strategy

Search strategy in Pubmed

Search number	Query
24	#20 AND #23
23	#21 OR #22
22	"random*"[Text Word] OR "blind*"[Text Word] OR "placebo"[tiab] OR "alllocat*"[tiab] OR "control"[tiab] OR "trial*"[ti] OR volunteer*[tiab] OR groups[tiab]
21	"Randomized Controlled Trial"[Publication Type] OR "Controlled Clinical Trial"[Publication Type] OR "Clinical Trial"[Publication Type] OR "Randomized Controlled Trials as Topic"[MeSH Terms] OR "Controlled Clinical Trials as Topic"[MeSH Terms] OR "Clinical Trials as Topic"[MeSH Terms]
20	#18 OR #19
19	mouth exercise*[Title/Abstract] OR mouth training[Title/Abstract] OR oral exercise*[Title/Abstract] OR oral training[Title/Abstract]
18	#8 AND #17
17	#9 OR #10 OR #11 OR #12 OR #13 OR #14 OR #15 OR #16
16	isometric*[Title/Abstract]
15	strengthening[Title/Abstract]
14	Strength* Program*[Title/Abstract]
13	Muscle Stretching Exercises[MeSH Terms]
12	training[Title/Abstract]
11	Resistance Training[MeSH Terms]
10	exercise*[Title/Abstract]
9	Exercise therapy[MeSH Terms]
8	#1 OR #2 OR #3 OR #4 OR #5 OR #6 OR #7
7	Oral-muscle[Text Word]
6	Oral-facial[Text Word]
5	Oralfacial[Text Word]
4	Lingua*[Text Word]
3	Lingual Frenum[MeSH Terms]

2	Tongue*[Text Word]
1	Tongue[MeSH Terms]

Search strategy in Embase

Search number	Query
#18	#14 AND #17
#17	#15 OR #16
#16	random*:ab,ti OR blind*:ab,ti OR placebo:ab,ti OR alllocat*:ab,ti OR control:ab,ti OR volunteer*:ab,ti OR groups:ab,ti OR trial*:ti,ab
#15	'randomized controlled trial'/exp OR 'controlled trial, randomized':ti,ab,kw OR 'randomised controlled study':ti,ab,kw OR 'randomised controlled trial':ti,ab,kw OR 'randomized controlled study':ti,ab,kw OR 'randomized controlled trial':ti,ab,kw OR 'trial, randomized controlled':ti,ab,kw OR 'controlled study'/exp OR 'control group study':ti,ab,kw OR 'control group trial':ti,ab,kw OR 'controlled study':ti,ab,kw OR 'controlled trial':ti,ab,kw OR 'controlled clinical trial'/exp OR 'clinical trial, controlled':ti,ab,kw OR 'controlled clinical comparison':ti,ab,kw OR 'controlled clinical drug trial':ti,ab,kw OR 'controlled clinical experiment':ti,ab,kw OR 'controlled clinical study':ti,ab,kw OR 'controlled clinical test':ti,ab,kw OR 'controlled clinical trial':ti,ab,kw
#14	#12 OR #13
#13	((mouth OR oral) NEAR/3 (exercise* OR training OR 'strength* program*')):ti,ab,kw
#12	#4 AND #11
#11	#5 OR #6 OR #7 OR #8 OR #9 OR #10
#10	exercise*:ab,ti OR training:ab,ti OR program:ab,ti OR 'strength* program*':ab,ti
#9	'stretching exercise'/exp OR 'muscle stretching exercises':ti,ab,kw OR 'stretching exercise':ti,ab,kw OR 'stretching exercises':ti,ab,kw
#8	'resistance training'/exp OR 'resistance exercise':ti,ab,kw OR 'resistance exercise training':ti,ab,kw OR 'resistance training':ti,ab,kw OR 'resistance-type exercise':ti,ab,kw OR 'resistance-type training':ti,ab,kw OR 'strength training':ti,ab,kw OR 'strength-type exercise':ti,ab,kw OR 'strength-type training':ti,ab,kw
#7	'isometric exercise'/exp OR 'exercise, isometric':ti,ab,kw OR 'isometric endurance':ti,ab,kw OR 'isometric endurance test':ti,ab,kw OR 'isometric exercise':ti,ab,kw OR 'isometric training':ti,ab,kw

#6	'muscle exercise'/exp OR 'muscle endurance':ti,ab,kw OR 'muscle exercise':ti,ab,kw OR 'muscle exertion':ti,ab,kw OR 'muscular exercise':ti,ab,kw OR 'muscular exertion':ti,ab,kw
#5	'muscle training'/exp OR 'muscle strengthening':ti,ab,kw OR 'muscle training':ti,ab,kw OR 'myofunctional therapy':ti,ab,kw OR 'training, muscle':ti,ab,kw
#4	#1 OR #2 OR #3
#3	'oral muscle':ab,ti
#2	oralfacial:ab,ti OR 'oral facial':ab,ti
#1	'tongue'/exp OR 'lingua':ti,ab,kw OR 'lingual':ti,ab,kw OR 'tongue':ti,ab,kw OR 'tongue pressure':ti,ab,kw OR 'tongue frenulum'/exp OR 'frenulum linguae':ti,ab,kw OR 'frenulum, lingual':ti,ab,kw OR 'frenulum, tongue':ti,ab,kw OR 'lingual frenulum':ti,ab,kw OR 'lingual frenum':ti,ab,kw OR 'oral frenulum':ti,ab,kw OR 'tongue frenulum':ti,ab,kw

Search strategy in Cochrane Library

ID	Search
#1	MeSH descriptor: [Tongue] explode all trees
#2	MeSH descriptor: [Lingual Frenum] explode all trees
#3	(Tongue* OR Lingua*):ti,ab,kw
#4	oralfacial:ti,ab
#5	oral NEAR/3 muscle
#6	#1 OR #2 OR #3 OR #4 OR #5
#7	MeSH descriptor: [Exercise Therapy] explode all trees
#8	MeSH descriptor: [Resistance Training] explode all trees
#9	MeSH descriptor: [Muscle Stretching Exercises] explode all trees
#10	(Strength* NEAR/2 Program*):ti,ab,kw
#11	(training OR exercise*):ti,ab,kw
#12	#7 OR #8 OR #9 OR #10 OR #11
#13	#6 AND #12
#14	((oral OR mouth) NEAR/3 (exercise* OR training)):ti,ab,kw (Word variations have been searched)
#15	#13 OR #14

Table S3. Characteristics of included studies

Study	Country	Participants	Intervention	Intervention protocol	Outcome assessment	Main findings
Lazarus et al., 2003	USA	31 healthy adults (23 females), mean age 26 yrs (20−29 yrs)	Intervention (*n* = 10): TSE using a tongue depressor; intervention (*n* = 11): TSE using the IOPI; control (*n* = 10): no exercise	Exercise in four directions: left, right, protrusion, and elevation; 2 sec on each repetition, 10 repetitions for each direction, 5 sets per day, 5 days a week for 4 weeks	Before and after 4 weeks: ATS (kPa) using the IOPI	Significantly higher posttest ATS in the TSE groups (73.1 ± 1.6) than in the control group (71.2 ± 5.4), $p = 0.04$
Clark 2012	USA	25 healthy adults (22 females), mean age 29.8 yrs (19−57 yrs)	Intervention (*n* = 5): TSE 100% 1RM using the IOPI; intervention (*n* = 5): Isotonic endurance training; intervention (*n* = 5): power training.; intervention (*n* = 5): speed training; control (*n* = 5): no exercise	Anterior tongue strengthening, 5 repetitions for a set, 5 sets per day, 3 days a week for 4 weeks	Before and after 4 weeks: ATS using the IOPI	Significantly increased ATS after TSE (82.6 ± 13.39) compared with that at baseline (65.8 ± 14.97)
Lazarus et al., 2014	USA	18 oropharyngeal cancer patients who underwent radiotherapy ± chemotherapy, mean age 61.9 yrs	Intervention (*n* = 8): traditional laryngeal excursion exercise and additional TSE using a tongue depressor; control (*n* = 10): traditional laryngeal excursion exercise	Exercise in four directions: left, right, protrusion, and elevation; 2 sec on each repetition, 10 repetitions for each direction, 5 sets per day, 5 days a week for 6 weeks	Before and after 10 weeks: ATS using the IOPI; OPSE scores; salivary flow measures	No significantly increased ATS after TSE (46.50 ± 16.50) compared with that at baseline (44.63 ± 13.39), $p = 0.571$
Park et al., 2015	Korea	29 stroke patients with dysphagia (16 females), mean age 66.6 yrs	Intervention (*n* = 15): traditional dysphagia therapy and additional TSE using the IOPI; control (*n* = 14): traditional dysphagia therapy for 30 min per day	Anterior and posterior tongue strengthening, 2 sec on each repetition, 10 repetitions for each portion, 5 sets per day, 5 days a week for 6 weeks	Before and after 6 weeks: ATS and PTS using the IOPI; VDS	Significantly increased ATS after TSE (20.73 ± 6.61) compared with that at baseline (18.93 ± 6.75), $p < 0.01$; significantly increased PTS after TSE (18.47 ± 4.09) compared with that at baseline (16.2 ± 4.69), $p < 0.01$

Kim et al. 2017	Korea	35 stroke patients with dysphagia (16 females), mean age 60.8 yrs	Intervention (*n* = 18): traditional dysphagia therapy and additional TSE using the air-filled bulb; control (*n* = 17): traditional dysphagia therapy 5 days a week for 4 weeks	Anterior and posterior tongue strengthening, 10 repetitions for each portion, 3 sets per day, 5 days a week for 4 weeks	Before and after 4 weeks: ATS and PTS using the IOPI; VDS; PAS	Significantly higher posttest ATS in the TSE groups (41.89 ± 9.54) than in the control group (32.53 ± 10.17), $p = 0.009$; significantly higher posttest PTS in the TSE groups (39.11 ± 7.8) than in the control group (31.41 ± 9.74), $p = 0.015$
Moon et al., 2017	Korea	16 stroke patients with dysphagia (5 females), mean age 65 yrs	Intervention (*n* = 8): traditional dysphagia therapy for 30 min and TSE using the IOPI for 30 min; control (*n* = 8): traditional dysphagia therapy for 30 min per day, 5 days a week for 4 weeks	Anterior tongue strengthening, 10 repetitions, 10 sec rest between each repetition, 30 min per day, 5 days a week for 4 weeks	Before and after 4 weeks: ATS using the IOPI; alternating motion rate; sequential motion rate	Significantly higher posttest ATS in the TSE groups (26.5 ± 2.7) than in the control group (21.8 ± 4.4), $p < 0.01$
Moon et al., 2018	Korea	16 stroke patients with dysphagia (9 females), mean age 62.8 yrs	Intervention (*n* = 8): traditional dysphagia therapy for 30 min and TSE using the IOPI for 30 min; control (*n* = 8): traditional dysphagia therapy for 60 min per day, 5 days a week for 8 weeks	Anterior and posterior tongue strengthening, 6 repetitions per set, 5 sets per day, 30 min per day, 5 days a week for 8 weeks	Before and after 8 weeks: ATS and PTS using the IOPI; MASA scale; SWAL-QOL	Significantly higher posttest ATS in the TSE groups (49.75 ± 5.26) than in the control group (35.5 ± 6.35), $p < 0.01$; significantly higher posttest PTS in the TSE groups (50.13 ± 4.32) than in the control group (32.13 ± 4.09), $p < 0.01$
Park et al., 2019	Korea	40 community-dwelling elderly aged over 65 (19 females), mean age 69.0 yrs	Intervention (*n* = 20): TSE using the TPS system; control (*n* = 20): no exercise	Isotonic exercise: 30 repetitions of contraction and relaxation, 3 times a day; isometric exercise: maintained contraction for 30 sec for a total of 3 sets	Before and after: ATS using the TPS system; tongue muscle thickness	Significantly higher posttest ATS in the TSE groups (43.92 ± 4.88) than in the control group (37.09 ± 3.36), $p = 0.001$

Park et al., 2019	Korea	30 healthy adults (15 females), mean age 24.8 yrs (21–35 yrs)	Intervention (*n* = 15): TSE; control (*n* = 15): no exercise	Isotonic exercise: 2 sec on each repetition, total 30 repetitions; isometric exercise: maintained for 10 sec for a total of 3 sets; 5 days a week for 6 weeks	Before and after 6 weeks: ATS using IOPI; tongue muscle thickness	Significantly increased ATS after TSE (57.66 ± 5.21) compared with that at baseline (52.5 ± 4.44), p = 0.002
Van den Steen et al., 2019	Belgium	60 healthy elderly aged 70 and over, living in the nursing home	Intervention (*n* = 15): TSE 100% 1RM using the IOPI; intervention (*n* = 16): TSE 80% 1RM using the IOPI; intervention (*n* = 16): TSE 60% 1RM using the IOPI; control (*n* = 13): LSE	Anterior and posterior tongue strengthening, 5 repetitions for each portion, 12 sets per day, 3 days a week for 8 weeks	Before and after 8 weeks: ATS and PTS using IOPI	Significantly increased ATS after TSE (59.4 ± 12.6) compared with that at baseline (36.9 ± 9.1), p < 0.001; significantly increased PTS after TSE (52.7 ± 12.3) compared with that at baseline (38.9 ± 12.3), p < 0.001
Lee et al., 2020	Korea	74 elderly aged 65 and over (62 females)	Intervention (*n* = 22): TSE using the IOPI; Intervention (*n* = 30): tongue-hold swallowing; control (*n* = 22): no exercise	Anterior and posterior tongue strengthening, 10 repetitions for each portion, 3 times per day, 3 days a week for 8 weeks	Before and after 8 weeks: ATS, PTS, LS using IOPI; salivary flow rate; OHIP-14 scale	Significantly increased ATS after TSE (45.50 ± 10.96) compared with that at baseline (40.64 ± 11.51), p = 0.007; no significant pre–post difference for PTS and LS
Lin et al., 2021	Taiwan	91 healthy adults (62 females), mean age 35.3 yrs (22–72 yrs)	Intervention (*n* = 44): TSE using the air-filled bulb; control (*n* = 47): no exercise	Anterior and posterior tongue strengthening, 30 repetitions for each portion, 5 days a week for 8 weeks	Before and after 8 weeks: ATS and PTS using IOPI	Significantly higher posttest ATS in the TSE groups (60.8 ± 1.6) than in the control group (55.1 ± 1.5), p = 0.011; significantly higher posttest PTS in the TSE groups (59.9 ± 1.3) than in the control group (55.1 ± 1.3), p = 0.01

[*]TPS system: TPS 100, Cybermedic Inc, Iksan, South Korea

Abbreviations: ATS, anterior tongue strength; IOPI, Iowa Oral Performance Instrument; kPa, kilopascals; LS, lip strength; LSE, lip-strengthening exercises; min, minute; MASA, Mann Assessment of Swallowing Ability; PAS, penetration aspiration scale; PTS, posterior tongue strength; RM, repetition maximum; sec, second; SWAL-QOL, Swallowing-Quality of Life; TSE, tongue-strengthening exercise; VDS, videofluoroscopic dysphagia scale; yrs, years

Table S4. Baseline tongue strength in each included study. ATS, anterior tongue strength; PTS, posterior tongue strength; SD, standard deviation; kPa, kilopascals

			Intervention			Control		
Study	Participants	Tongue strength	*n*	Baseline mean (kPa)	Baseline SD (kPa)	*n*	Baseline mean (kPa)	Baseline SD (kPa)
Lazarus et al., 2003	Healthy young adults	ATS	21	64.4	1.9	10	69.8	5.6
Clark 2012	Healthy young adults	ATS	5	65.8	15.0	5	66.8	13.2
Lazarus et al., 2014	Illness	ATS	8	44.6	13.4	10	49.3	10.5
Park et al., 2015	Illness	ATS	15	18.9	6.8	14	22.0	5.7
		PTS	15	16.2	4.7	14	17.3	4.3
Kim et al. 2017	Illness	ATS	18	32.7	10.8	17	29.7	10.4
		PTS	18	28.1	7.6	17	26.6	9.1
Moon et al., 2017	Illness	ATS	8	21.7	2.3	8	21.2	4.4
Moon et al., 2018	Illness	ATS	8	31.4	5.7	8	32.3	5.4
		PTS	8	28.5	4.8	8	29.8	4.4
Park et al., 2019	Healthy elderly	ATS	20	37.1	3.5	20	36.6	3.3
Park et al., 2019	Healthy young adults	ATS	15	52.5	4.4	15	53.8	3.0
Van den Steen et al., 2019	Healthy elderly	ATS	15	36.9	9.1	13	39.2	9.9
		PTS	15	30.2	8.3	13	34.6	8.7
Lee et al., 2020	Healthy elderly	ATS	22	40.6	11.5	22	39.1	12.9
		PTS	22	41.5	11.2	22	35.6	15.3
Lin et al., 2021	Healthy adults	ATS	44	60.2	11.8	47	53.2	15.7
		PTS	44	56.5	12.7	47	50.6	12.3

Table S5. Training protocols of included studies

Study	Device	Exercise	Time	Repetition	Number of sets per day	Frequency (days/week)	Duration (weeks)
Lazarus et al., 2003	Tongue depressor and IOPI	left, right, protrusion, and elevation	2 sec	10 for each direction	5	5	4
Clark 2012	IOPI	Anterior tongue	NA	5	5	3	4
Lazarus et al., 2014	Tongue depressor	left, right, protrusion, and elevation	2 sec	10 for each direction	5	5	6
Park et al., 2015	IOPI	Anterior and posterior tongue	2 sec	10 for each portion	5	5	6
Kim et al. 2017	IOPI	Anterior and posterior tongue	NA	10 for each portion	3	5	4
Moon et al., 2017	IOPI	Anterior tongue	NA	10	30 min per day	5	4
Moon et al., 2018	IOPI	Anterior and posterior tongue	NA	6	5	5	8
Park et al., 2019, aged 65 years and over	TPS system*	Isotonic and isometric exercise	NA	30	3	NA	NA
Park et al., 2019, aged under 65 years	No device	Isotonic and isometric exercise	2	30	NA	5	6
Van den Steen et al., 2019	IOPI	Anterior and posterior tongue	NA	5 for each portion	12	3	8
Lee et al., 2020	IOPI	Anterior and posterior tongue	NA	10 for each portion	3	3	8
Lin et al., 2021	IOPI	Anterior and posterior tongue	10 sec	30 for each portion	1	5	8

*TPS system: TPS 100, Cybermedic Inc, Iksan, South Korea

Abbreviations: IOPI, Iowa Oral Performance Instrument; NA, not available; sec, second

Table S6. Revised Cochrane risk-of-bias tool (RoB 2.0) for quality assessment of included RCTs

Domain1	Risk of bias arising from the randomization process	
Domain2	Risk of bias due to deviations from the intended interventions	
Domain3	Risk of bias due to missing outcome data	
Domain4	Risk of bias in measurement of the outcome	
Domain5	Risk of bias in selection of the reported result	
Overall risk of bias	Low risk of bias	The study is judged to be at low risk of bias for all domains for this result.
	Some concerns	The study is judged to raise some concerns in at least one domain for this result, but not to be at high risk of bias for any domain.
	High risk of bias	The study is judged to be at high risk of bias in at least one domain for this result. Or<br>The study is judged to have some concerns for multiple domains in a way that substantially lowers confidence in the result.

	Domain 1	Domain 2	Domain 3	Domain 4	Domain 5	Overall risk
Lazarus et al., 2003	Some concern	Low risk	Low risk	Low risk	Some concern	Some concern
Clark 2012	Low risk	Low risk	Low risk	Low risk	Some concern	Some concern
Lazarus et al., 2014	Low risk	Low risk	Low risk	Low risk	Some concern	Some concern
Park et al., 2015	Some concern	Low risk	Low risk	Low risk	Low risk	Some concern
Kim et al. 2017	Low risk	Low risk	Low risk	Low risk	Some concern	Some concern
Moon et al., 2017	Some concern	Low risk	Low risk	Low risk	Some concern	Some concern
Moon et al., 2018	Low risk	Low risk	Low risk	Low risk	Some concern	Some concern
Park et al., 2019; elderly	Some concern	Low risk	Low risk	Low risk	Some concern	Some concern
Park et al., 2019; young adults	Some concern	Low risk	Low risk	Low risk	Some concern	Some concern
Van den Steen et al., 2019	Some concern	Low risk	Low risk	Low risk	Some concern	Some concern
Lee et al., 2020	Some concern	Low risk	Low risk	Low risk	Low risk	Some concern
Lin et al., 2021	Some concern	Low risk	Low risk	Low risk	Some concern	Some concern

Figure S1. Forest plot of pooled anterior tongue strength after the intervention, comparing the tongue strengthening exercise group and the control group among the healthy participants (subgroup analysis by the age). MD, mean difference; CI, confidence interval.

Study	Experimental Total	Experimental Mean	Experimental SD	Control Total	Control Mean	Control SD	MD	95%-CI	Weight
Age = Both									
Lin et al., 2021	44	60.80	1.6000	47	55.10	1.5000	5.70	[5.06; 6.34]	32.7%
Random effects model	44			47			5.70	[5.06; 6.34]	32.7%
Heterogeneity: not applicable									
Age = Elderly									
Park et al., 2019, aged 65 years and over	20	43.92	4.8800	20	37.09	3.3600	6.83	[4.23; 9.43]	20.5%
Van den Steen et al., 2019	15	59.40	12.6000	13	44.50	11.7000	14.90	[5.89; 23.91]	3.8%
Lee et al., 2020	22	45.50	10.9600	22	38.36	11.1200	7.14	[0.62; 13.66]	6.6%
Random effects model	57			55			8.01	[4.39; 11.64]	30.8%
Heterogeneity: $I^2 = 30\%$, $\tau^2 = 3.6586$, $p = 0.24$									
Age = Young									
Lazarus et al., 2003	21	73.10	1.6000	10	71.20	5.4000	1.90	[-1.52; 5.32]	15.8%
Clark 2012	5	82.60	13.3900	5	73.60	10.0600	9.00	[-5.68; 23.68]	1.5%
Park et al., 2019, aged under 65 years	15	57.66	5.2100	15	54.72	1.9500	2.94	[0.12; 5.76]	19.1%
Random effects model	41			30			2.66	[0.51; 4.81]	36.5%
Heterogeneity: $I^2 = 0\%$, $\tau^2 = 0$, $p = 0.62$									
**Random effects model**	**142**			**132**			**5.30**	**[3.44; 7.15]**	**100.0%**
Heterogeneity: $I^2 = 55\%$, $\tau^2 = 2.6441$, $p = 0.04$									
Residual heterogeneity: $I^2 = 0\%$, $p = 0.43$									

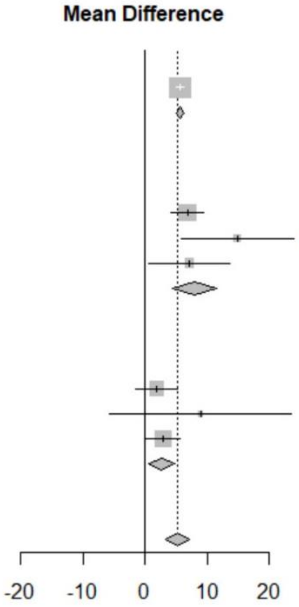

Figure S2. Meta-regression bubble plot of the correlation between effect of tongue strengthening exercise on anterior tongue strength and baseline anterior tongue strength. Each bubble represents a study and bubble size represents the sample size of the study.

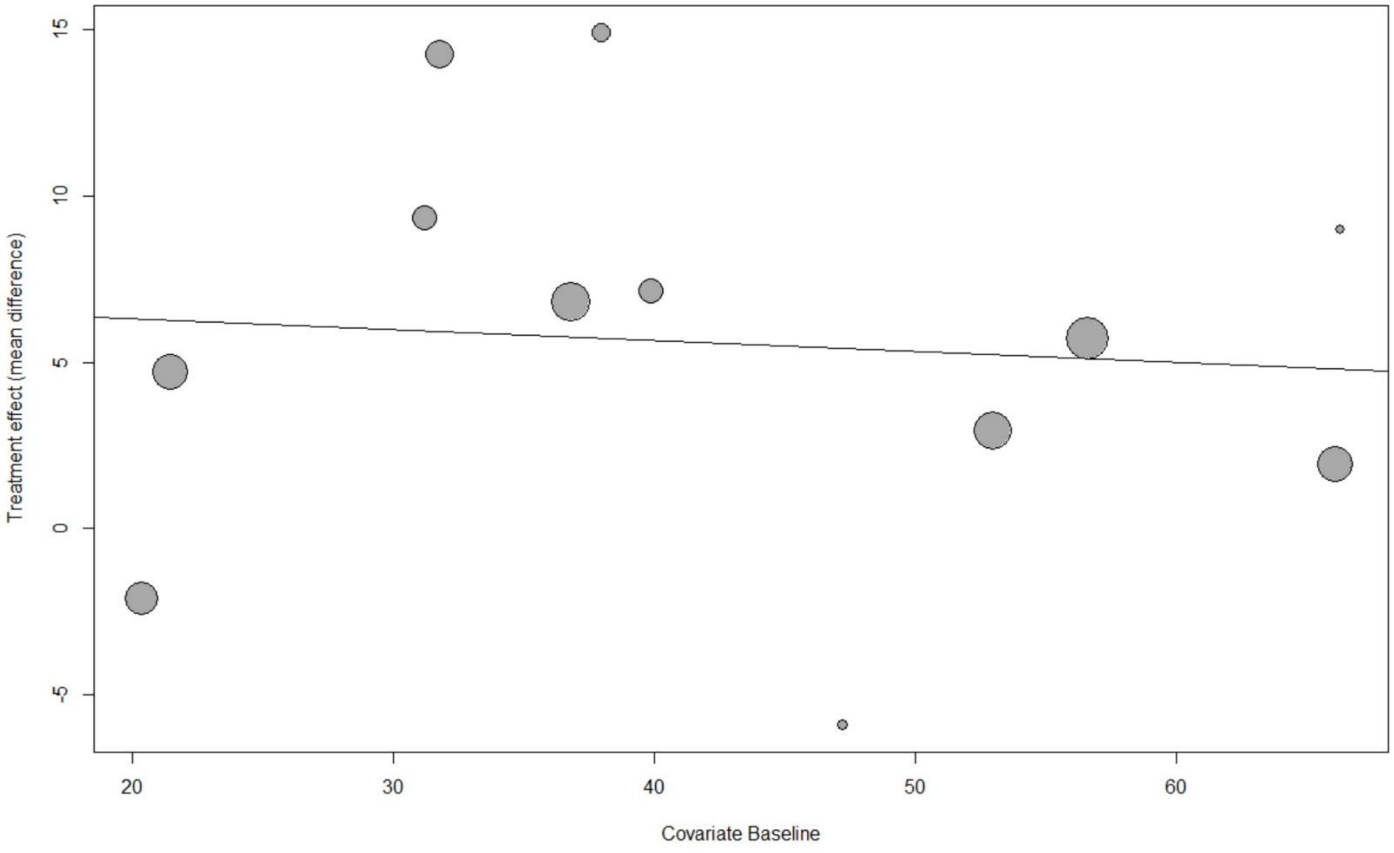

Figure S3. Meta-regression bubble plot of the correlation between effect of tongue strengthening exercise on anterior tongue strength and the total number of repetitions per day. Each bubble represents a study and bubble size represents the sample size of the study.

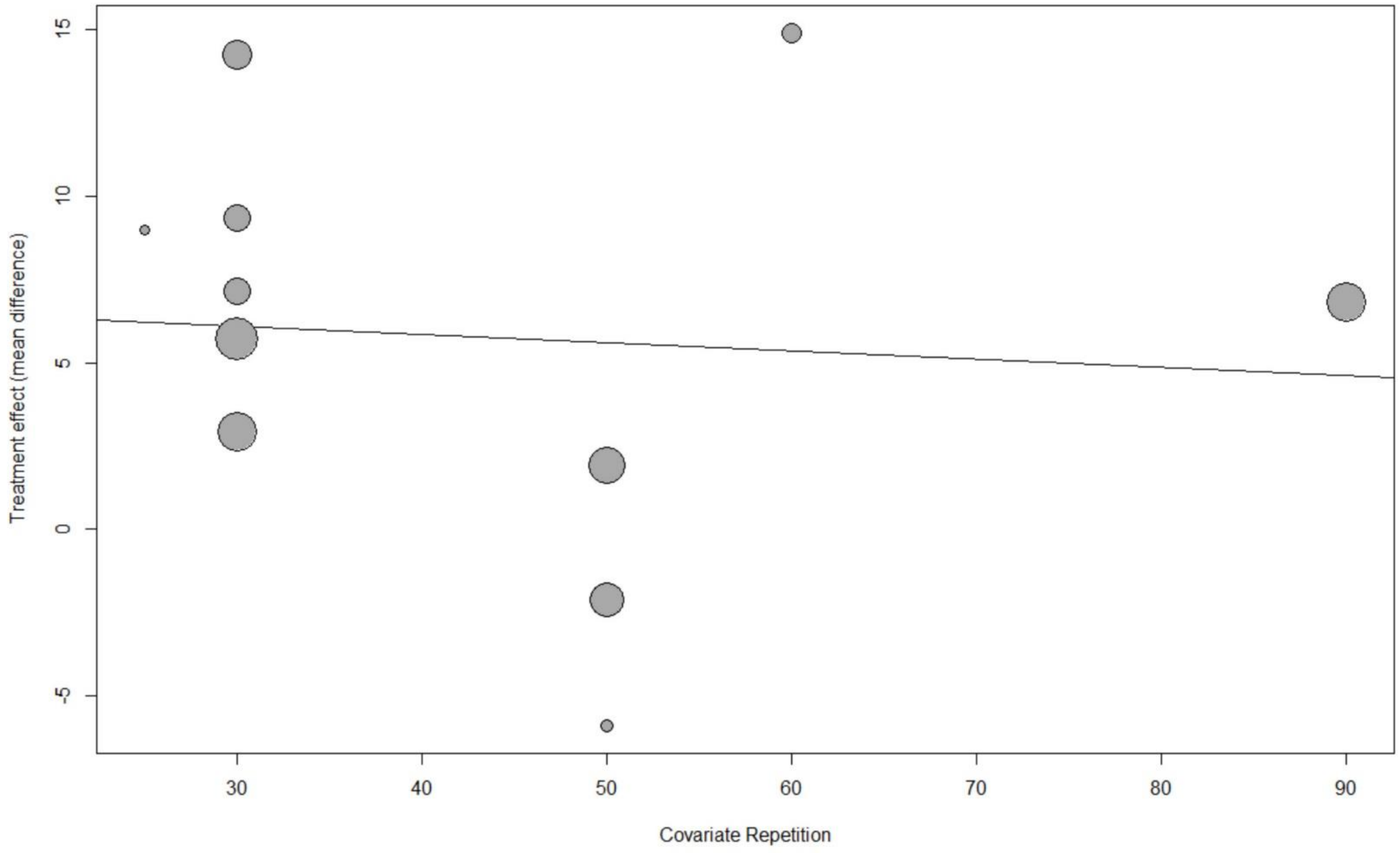

Figure S4. The Funnel plot of the standard error by mean difference of the studies included in the meta-analysis. Egger's test, slope = 5.59, p = 0.89

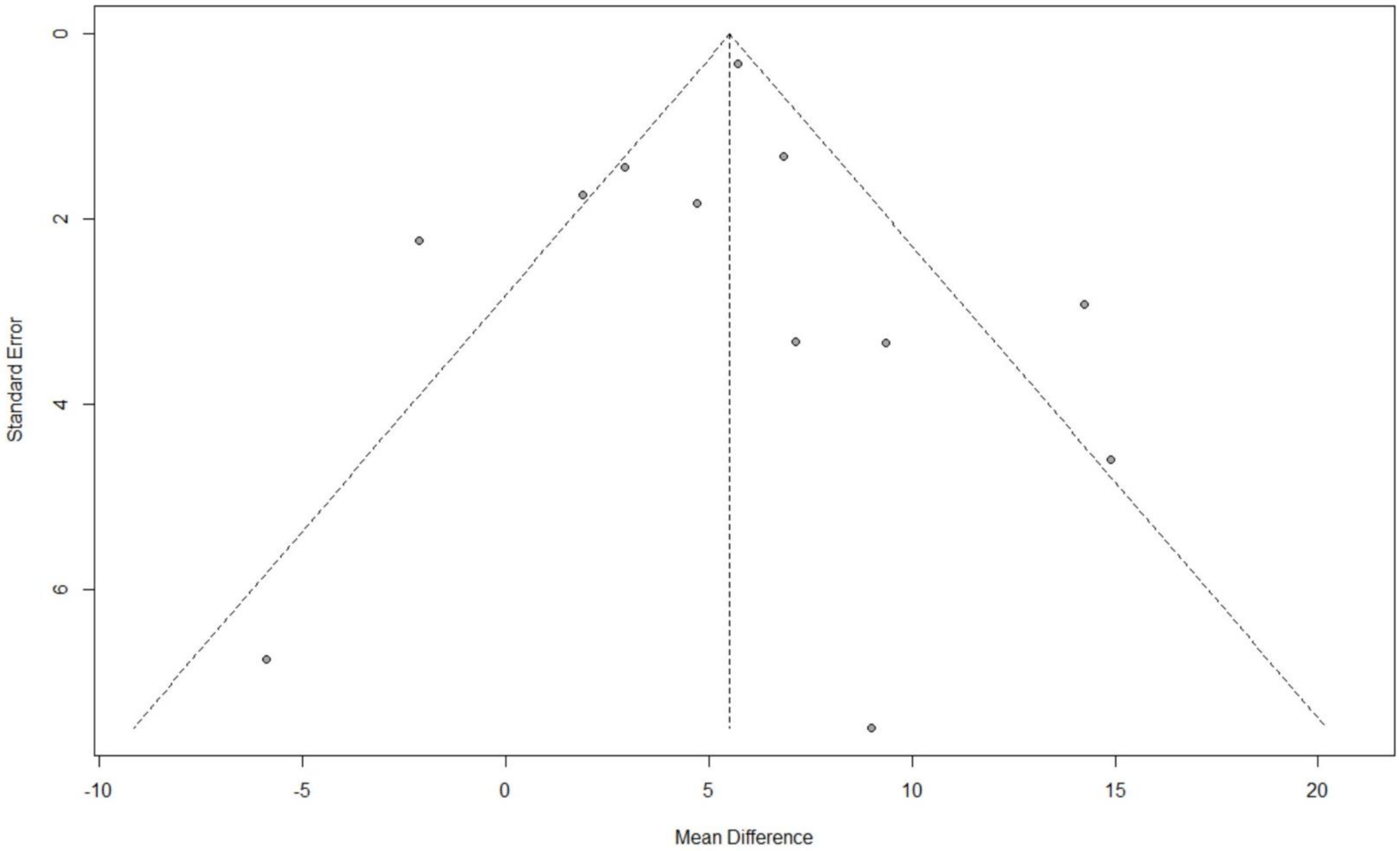

Figure S5. Sensitivity analyses of tongue strengthening exercise on anterior tongue strength by omitting each study. MD, mean difference; CI, confidence interval.

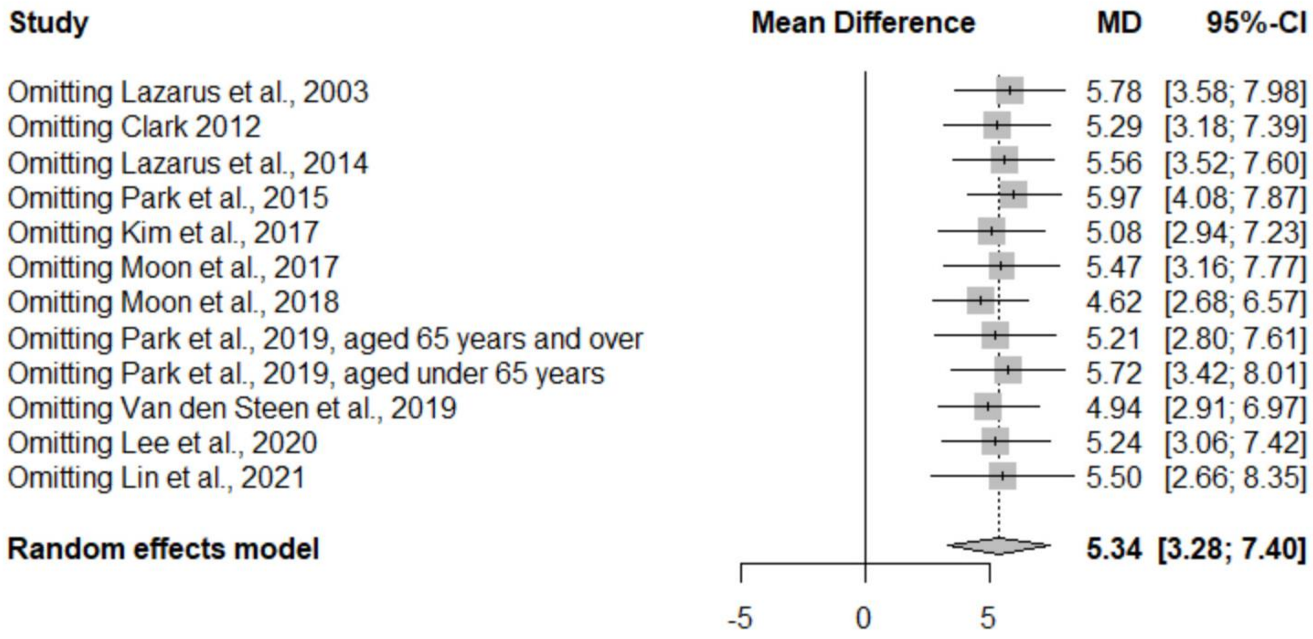

Figure S6. Meta-regression bubble plot of the correlation between effect of tongue strengthening exercise on posterior tongue strength and the duration of intervention. Each bubble represents a study and bubble size represents the sample size of the study.

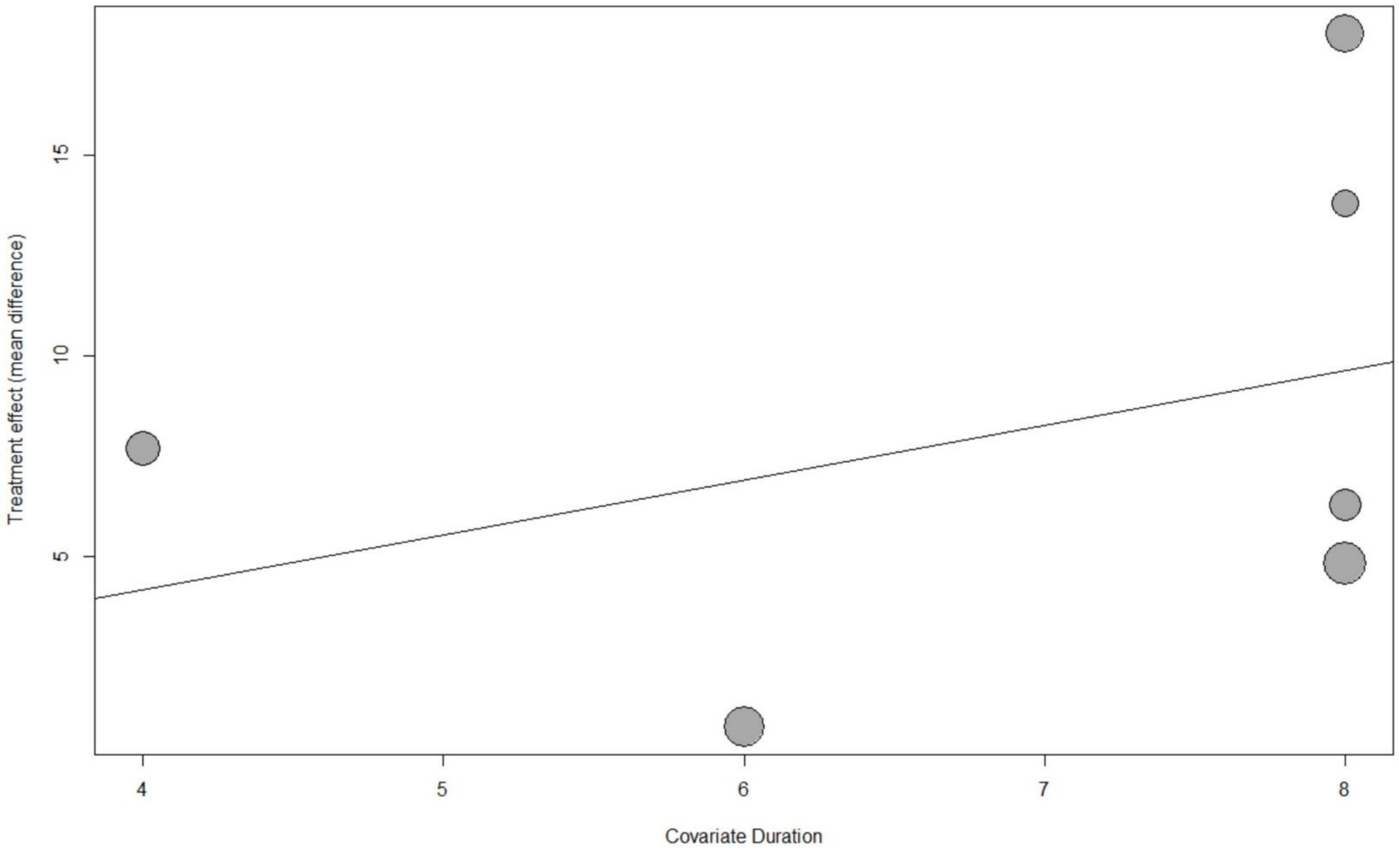

Figure S7. Meta-regression bubble plot of the correlation between effect of tongue strengthening exercise on posterior tongue strength and baseline posterior tongue strength. Each bubble represents a study and bubble size represents the sample size of the study.

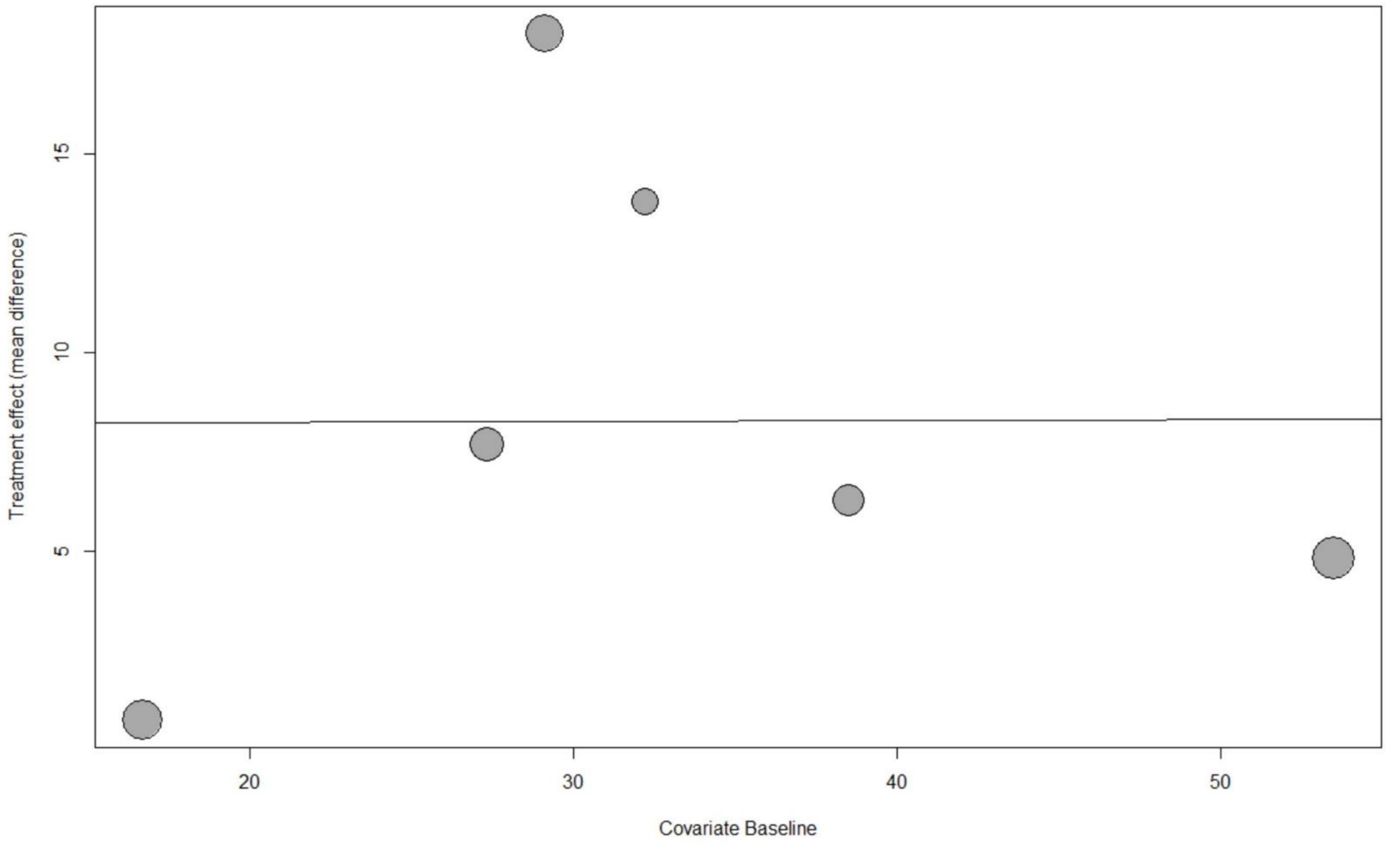

Figure S8. Meta-regression bubble plot of the correlation between effect of tongue strengthening exercise on posterior tongue strength and the total number of repetitions per day. Each bubble represents a study and bubble size represents the sample size of the study.

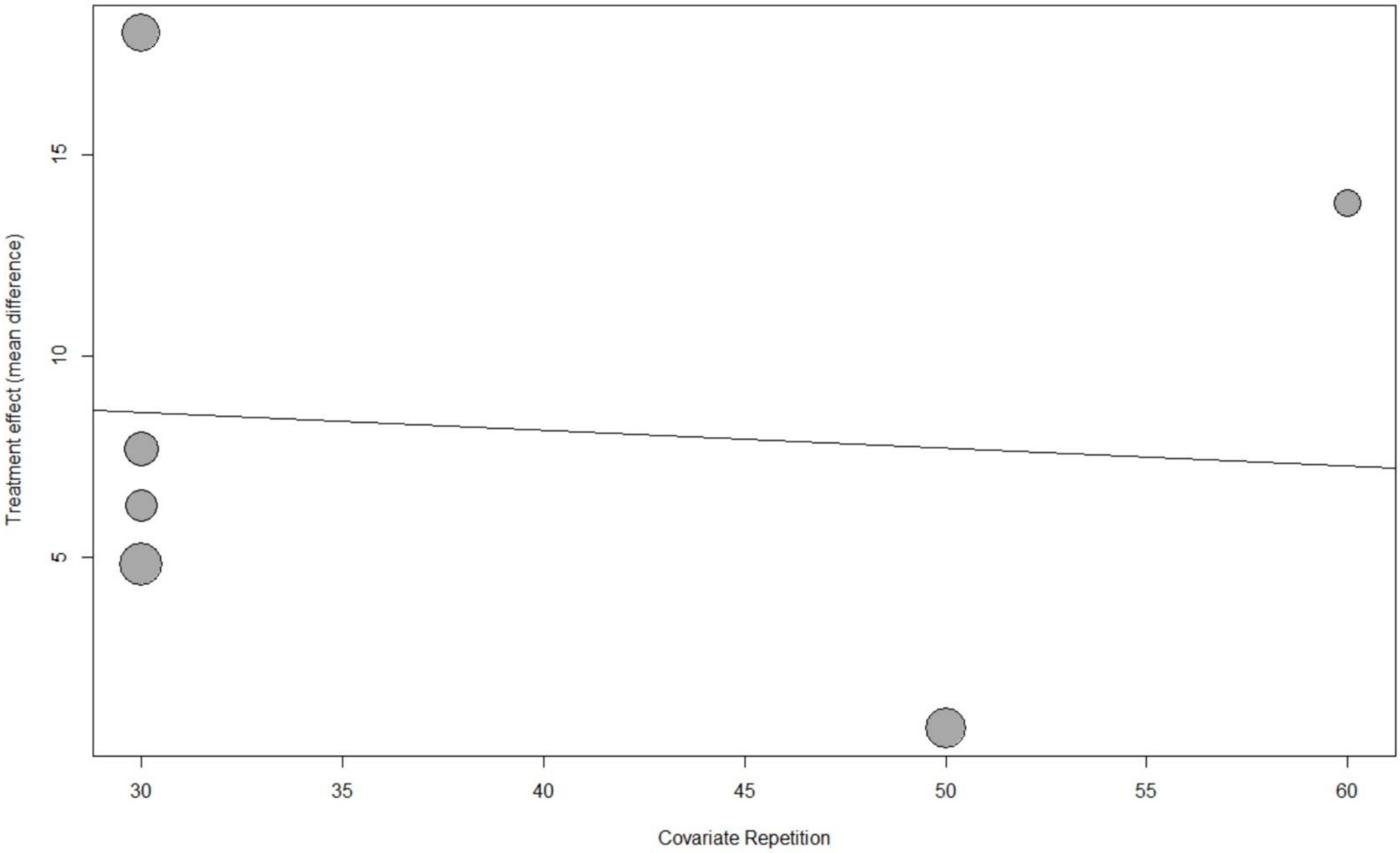

Figure S9. The Funnel plot of the standard error by mean difference of the studies included in the meta-analysis. Egger's test, slope = 4.32, $p = 0.38$

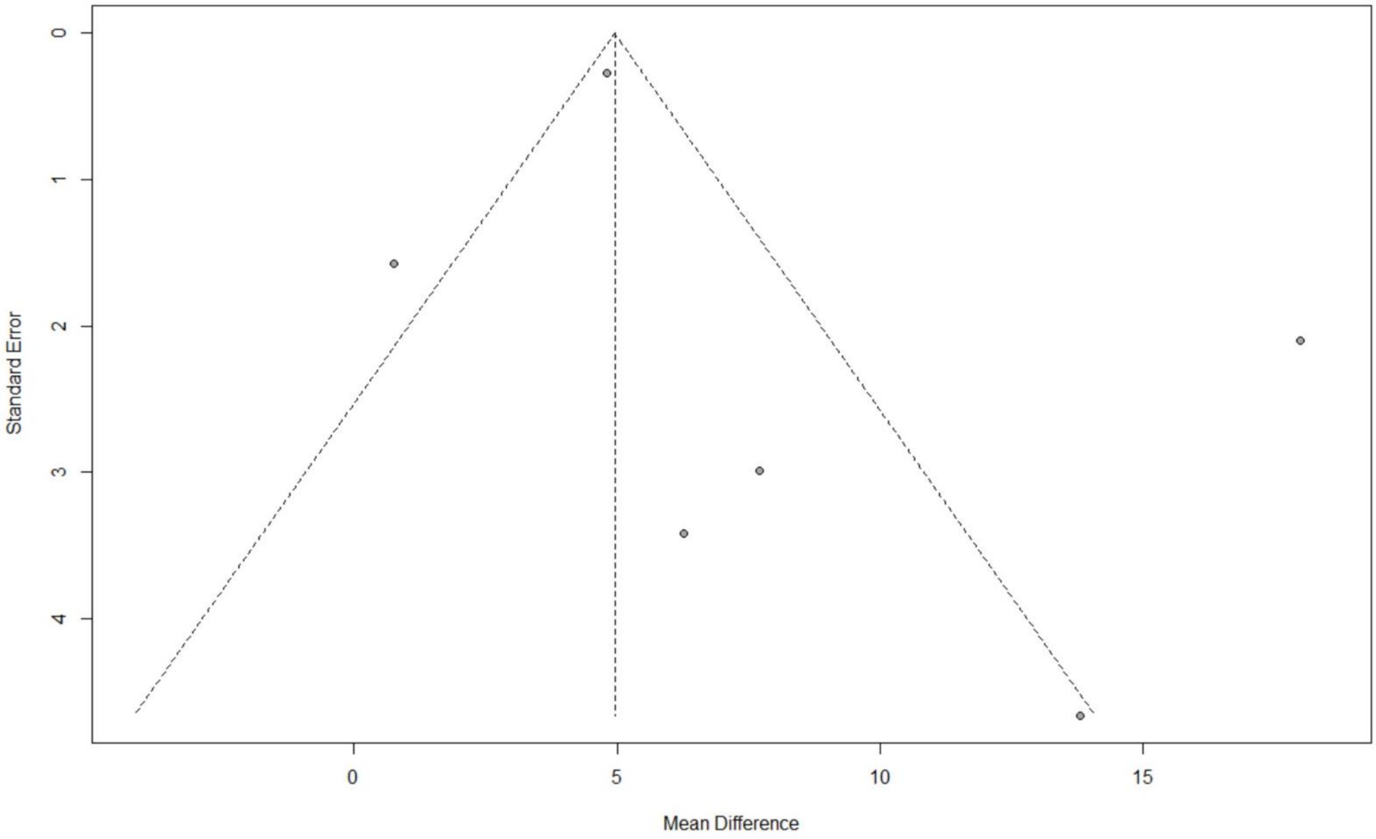

Figure S10. Sensitivity analyses of tongue strengthening exercise on posterior tongue strength by omitting each study. MD, mean difference; CI, confidence interval.

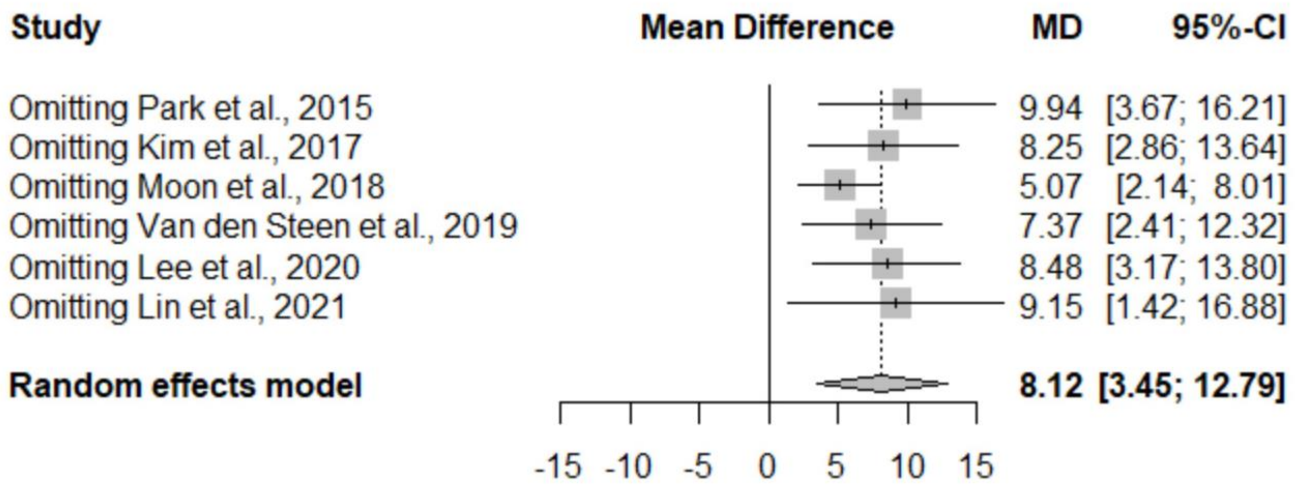

Supplement: Supplementary file 1 — Supplementary Information. [file 41598_2022_14335_MOESM1_ESM.pdf]
